# Total cholesterol, high-density lipoprotein, and glucose (CHG) index and diabetic retinopathy in middle-aged and elderly Chinese adults with diabetes: a cross-sectional study

**DOI:** 10.3389/fendo.2025.1682279

**Published:** 2026-01-22

**Authors:** Yingpin Cao, Yuqin He, Jiaqian Zhu, Yong Han

**Affiliations:** 1 Hunan Provincial University Key Laboratory of the Fundamental and Clinical Research on Neurodegenerative Diseases, Changsha Medical University, Changsha, Hunan, China; 2 Department of Ophthalmology, Shenzhen People’s Hospital (The First Affiliated Hospital, Southern University of Science and Technology; The Second Clinical Medical College, Jinan University), Shenzhen, Guangdong, China; 3 Hunan Provincial University Key Laboratory of the Fundamental and Clinical Research on Neurodegenerative Diseases, School of Medical Imaging, Changsha Medical University, Changsha, Hunan, China; 4 Department of Neurology, The First Affiliated Hospital of Shenzhen University, Shenzhen Second People’s Hospital, Shenzhen University, Shenzhen, Guangdong, China; 5 Department of Emergency, Shenzhen Second People’s Hospital, The First Affiliated Hospital of Shenzhen University, Shenzhen, Guangdong, China

**Keywords:** complications of diabetes, diabetic retinopathy, discriminative ability, insulin resistance, total cholesterol, high-density lipoprotein, and glucose index

## Abstract

**Objective:**

Evidence regarding the association between the total cholesterol, high-density lipoprotein, and glucose (CHG) index and diabetic retinopathy (DR) remains limited. This study aimed to explore the relationship between CHG and the prevalence of DR and evaluate its discriminative ability for DR.

**Methods:**

This cross-sectional study analyzed data from 1,909 individuals with diabetes mellitus (DM), aged 45–90 years, whose information was collected between August and December 2011. To determine the association between CHG and DR, binary logistic regression models were employed. The discriminative ability of CHG for DR was assessed using receiver operating characteristic (ROC) analysis. Additionally, a series of sensitivity analyses and subgroup analyses were conducted.

**Results:**

After multivariable adjustment, binary logistic regression analysis showed that each 0.1−unit increase in CHG was associated with a 14.2% higher prevalence of DR (OR = 1.142; 95% CI: 1.081–1.206). Additionally, CHG demonstrated the highest area under the curve (AUC) for discriminating DR (0.6673, 95% CI: 0.6287–0.7059), outperforming triglyceride-glucose body mass index (TyG-BMI, 0.4958, 95% CI: 0.4548–0.5368), triglyceride to high-density lipoprotein cholesterol ratio (THR, 0.5118, 95% CI: 0.4704–0.5532), and triglyceride-glucose index (TyG, 0.5720, 95% CI: 0.5314–0.6125). Sensitivity analysis and subgroup analyses further confirmed the reliability of these results.

**Conclusion:**

This study demonstrates that elevated CHG is independently and positively associated with DR in adults with DM. Moreover, CHG shows a certain discriminative ability for DR and may have potential utility in DR assessment.

## Introduction

Diabetic retinopathy (DR) is a common and specific retinal microvascular complication in individuals with diabetes mellitus (DM), and one of the leading causes of vision impairment and blindness among individuals with DM worldwide (1, 2). With the rapid growth of the global population with DM, the burden of DR continues to rise, posing significant public health challenges, particularly in low- and middle-income countries (3–5). Its prevalence and severity vary significantly between developed and developing countries, mainly due to differences in healthcare accessibility, awareness, and screening practices (6, 7). However, in developing regions, delayed diagnosis and limited availability of ophthalmologic services often lead to advanced disease at the first presentation (8, 9). For instance, a recent study demonstrated that 12.5% of individuals with DM in a developing country presented with proliferative DR at their initial visit, indicating a considerable delay in detection and intervention (9). These disparities underscore the urgent need for simple, accessible, and cost-effective screening tools that can be implemented even in low-resource primary care settings to enable early DR detection and timely referral.

In recent years, a novel composite index termed the total cholesterol, high-density lipoprotein cholesterol, and glucose (CHG) index, has been proposed by Amin Mansoori et al. as an indicator of metabolic health and cardiometabolic risk (10). The CHG index is derived from three readily accessible laboratory parameters: total cholesterol (TC), high-density lipoprotein cholesterol (HDL-c), and fasting plasma glucose (FPG). Notably, its ease of calculation from routine blood tests makes it particularly suitable for implementation in primary care settings with limited resources. Previous studies have demonstrated the utility of the CHG index in predicting DM and cardiovascular events (10, 11).

However, research on the relationship between CHG and DR is currently quite limited. Only one small-scale cross-sectional study by Merve et al., involving 175 individuals with DM, reported significantly elevated CHG levels in those with DR. Nevertheless, the multivariable logistic regression analysis failed to establish a statistically significant association, likely due to insufficient statistical power resulting from the limited sample size (12). Therefore, this study aimed to investigate the association between CHG and DR using a large-sample cross-sectional design, and to evaluate its discriminative ability for identifying DR.

## Methods

### Study design and data source

This study utilized a cross-sectional approach, drawing on baseline data derived from a multicenter, prospective cohort recruited across various sites in China. The primary purpose of the underlying cohort was to assess potential associations between disturbances in glucose metabolism and elevated cancer risk within the Chinese population (13). The dataset used for this secondary analysis was derived from the study by Wang et al. This resource is publicly available in the PLOS database at the following URL: https://journals.plos.org/plosone/article?id=10.1371/journal.pone.0166597 (13). Comprehensive details regarding the study population and methodology are available in the original publication, “Cutoff Point of HbA1c for Diagnosis of Diabetes Mellitus in Chinese Individuals”(DOI: 10.1371/journal.pone.0166597) (13). This article is published under a Creative Commons Attribution-Noncommercial (CC BY-NC 4.0) license, which permits non-commercial sharing, redistribution, modification, and the creation of derivative works based on the original material (13). The source study received ethical approval from the Ethics Committee of Ruijin Hospital, Shanghai Jiao Tong University School of Medicine, and all participants provided formal written informed consent (13). As with many previous studies, this secondary analysis did not require reapplication for ethical approval.

### Study population

**Figure 1:**
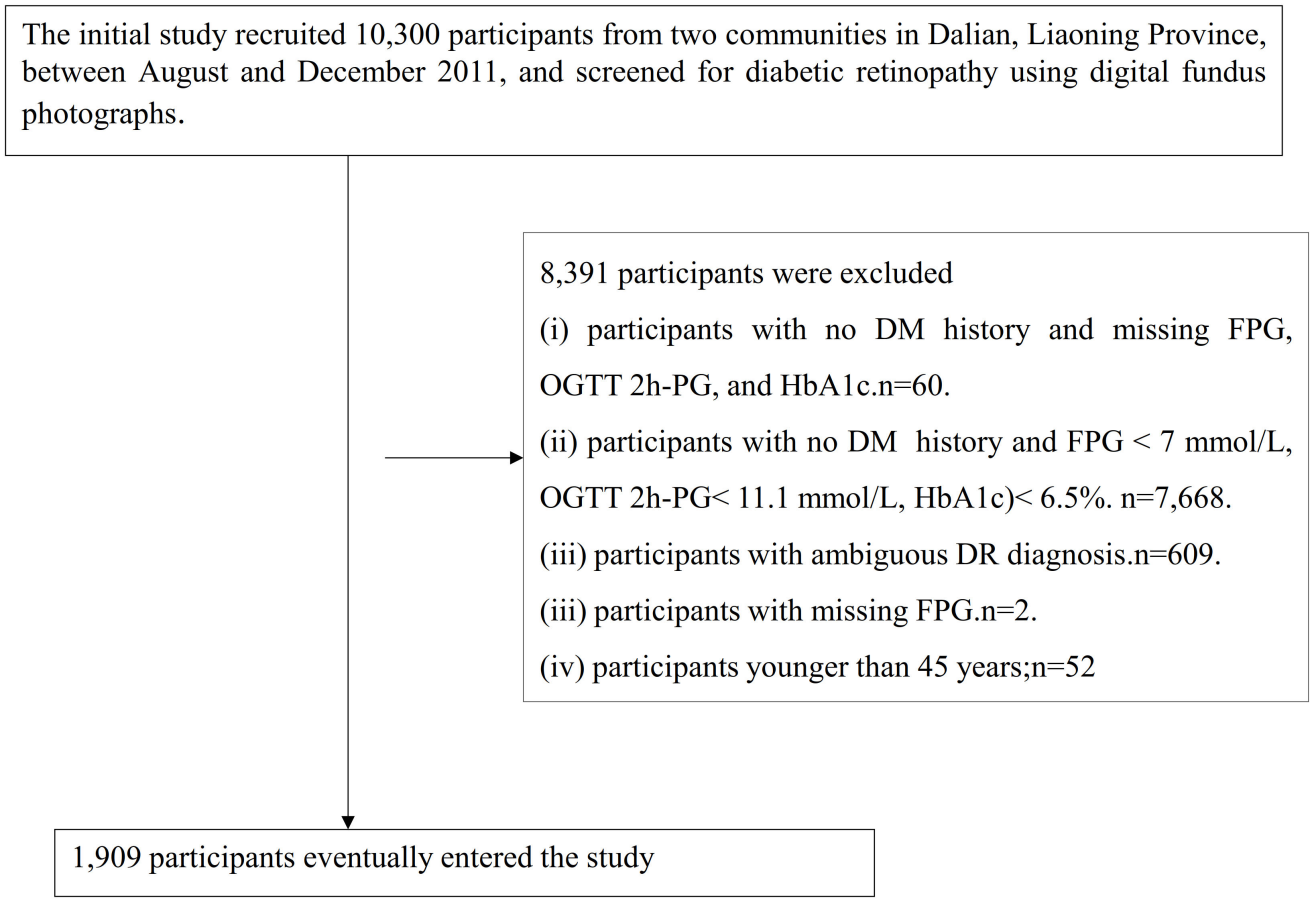
Flowchart illustrating the study participants.

The original study recruited a total of 10,300 participants aged 40 to 90 years from two communities in Dalian, Liaoning Province, between August and December 2011 (13). Participants were screened for DR using digital fundus photographs (CRNON CR6-45NM non-mydriatic ophthalmoscope digital camera produced by Canon, Japan) (13). Based on the objectives of this study, the following additional exclusion criteria were applied: (i) 60 participants with ambiguous DM diagnosis: those with no DM history and missing fasting plasma glucose (FPG), oral glucose tolerance test 2-hour glucose (OGTT 2h-PG), and hemoglobin A1c (HbA1c); (ii) 7,668 participants who could not be diagnosed with DM: those with no DM history and FPG < 7 mmol/L, OGTT 2h-PG < 11.1 mmol/L, and HbA1c < 6.5%; (iii) 609 participants with ambiguous DR diagnosis; (iv) 2 participants with missing FPG; (v) According to the age classification criteria for middle-aged and older adults defined in the Report on Chinese Residents’ Nutrition and Chronic Diseases issued by the National Health Commission of the People’s Republic of China, 52 participants aged below 45 years were excluded. Finally, the study included 1,909 participants. The participant selection process is illustrated in **Figure 1**.

### Variables

#### Total cholesterol, high-density lipoprotein, and glucose (CHG) index

The CHG index was evaluated as a continuous measure. The index was computed according to the formula: CHG = Ln [TC × FPG/(2 × HDL-c)], where TC is total cholesterol (mg/dL), FPG is fasting plasma glucose (mg/dL), and HDL-c is high-density lipoprotein cholesterol (mg/dL) (10, 12). The coefficient “2” in the denominator is an integral component of the original CHG formula proposed by Mansoori et al., designed to achieve numerical standardization by balancing the magnitude of the numerator and denominator. This standardization prevents statistical instability after logarithmic transformation and is analogous to the approach used in established metabolic indices such as the TyG index ln[(TG × FPG/2)], where the coefficient similarly optimizes the numerical range and discriminatory performance of the composite index (10, 14). Importantly, in the CHG formula, factor 2 is used for numerical scaling and stability after logarithmic transformation and does not imply any pathophysiologic weighting of HDL-c relative to TC or FPG.

#### Outcome measures

In our study, the outcome variable was DR (dichotomous variable: 1=DR, 0=non-DR). The assessment of DR was conducted by collecting digital fundus photographs from both eyes of each participant (13). All images were independently evaluated by two qualified ophthalmologists who were blinded to the participants’ blood glucose and HbA1c levels. In cases of disagreement between the two ophthalmologists, an endocrinologist was invited to participate in the discussion (13).

#### Covariates

The selection of covariates for this analysis was based on clinical expertise and existing literature. The included covariates were as follows: (i) continuous variables—age, FPG, OGTT 2h-PG, HbA1c, height, weight, body mass index (BMI), waist circumference (WC), HDL-c, low-density lipoprotein cholesterol (LDL-c), TC, triglycerides (TG), Uric Acid (UA), Systolic Blood Pressure (SBP), and Diastolic Blood Pressure (DBP); (ii) categorical variable—Sex.

### Data collection and definitions

In the preliminary study, baseline information was collected by trained researchers using standardized questionnaires, which included demographic characteristics and history of DM (13). Body measurements, including height, weight, hip circumference, and waist circumference, were recorded for each participant (13). Blood pressure was measured three times consecutively on the right arm of the subjects, with one-minute intervals between each measurement, and the average of the three readings was used for analysis (13). After the participants fasted for at least 8 hours, venous blood samples were collected to determine the levels of HDL-c, FPG, TG, HbA1c, UA, TC, and LDL-c. Participants without a history of DM underwent a 75 g OGTT, and venous blood samples were collected two hours after the test to measure 2h-PG (13). It should be noted that the original study did not provide routine renal function tests (e.g., serum creatinine, estimated glomerular filtration rate, blood urea nitrogen), hematologic variables (e.g., hemoglobin, hematocrit), or lifestyle information (e.g., smoking, drinking, physical activity).

The definitions of BMI, triglyceride-glucose index (TyG), triglyceride-glucose body mass index (TyG-BMI), waist-to-hip ratio (WHR), and triglyceride-to-high-density lipoprotein cholesterol ratio (THR) were as follows: BMI was defined by dividing body weight (kg) by height squared (m²). WHR was calculated by dividing the waist circumference (cm) by the hip circumference(cm) (13). The TyG index was defined as ln[FPG (mg/dL) ×TG (mg/dL)/2] (14). TyG-BMI was defined as the product of BMI and the TyG index (TyG-BMI = BMI × TyG) (15). THR was defined as the ratio of triglycerides to high-density lipoprotein cholesterol. The definition of DM in this study was as follows: 1. Patients who self-reported a history of DM**;** 2. Individuals without a previous history of DM but who met any of the following criteria: FPG ≥7.0 mmol/L, OGTT 2h-PG ≥11.1 mmol/L after an OGTT, or HbA1c ≥6.5%, were diagnosed with DM (13).

### Missing data processing

Missing data is common in observational studies and often difficult to avoid. In the secondary analysis, several variables had missing values, including HbA1c (1, 0.05%), 2h-PG (4, 0.21%), TG (4, 0.21%), and UA (7, 0.37%). To minimize the bias that missing data might cause, we used multiple imputation to fill in the missing values in the dataset (16, 17). This method was based on linear regression and was executed over 10 iterations, incorporating the following variables: age, 2h-PG, HbA1c, height, weight, BMI, WC, LDL-c, TG, UA, SBP, and DBP. The missing values were assumed to be missing at random (MAR), in accordance with accepted analytical standards (17).

### Statistical analysis

To conduct intergroup comparisons, the baseline characteristics of the study subjects were stratified according to the quartiles of CHG. Continuous variables conforming to a normal distribution were summarized as mean ± standard deviation (SD), whereas those with skewed distributions were reported as median and interquartile range (IQR). Categorical variables were presented as counts and percentages. For group comparisons, one-way analysis of variance (ANOVA) or the Kruskal-Wallis test was selected according to the distributional characteristics of continuous variables. The chi-square (χ²) test was used to assess differences between groups for categorical outcomes.

Univariate and multivariable binary logistic regression models were constructed to assess the association between CHG and the likelihood of DR. Prior to model construction, variance inflation factors (VIFs) were calculated for all candidate covariates to assess multicollinearity, with VIF > 10 being used as the threshold to indicate significant collinearity. In Model I, no covariate adjustment was made; in Model II, adjustments were made for DBP, BMI, age, WHR, sex, HbA1c, SBP, HIP, and TG. WC was excluded from the multivariable regression models because its VIF value exceeded 10, indicating substantial collinearity with other variables (see **Supplementary Table S1**). In addition, a generalized additive model (GAM) was used within the multivariable logistic regression framework to incorporate continuous covariates as smooth functions.

Previous studies have confirmed that TG, TC, and obesity are significantly related to the likelihood of DR (18, 19). To validate the stability of the findings, sensitivity analyses were performed by sequentially excluding patients with obesity (BMI ≥28 kg/m²), TG ≥150 mg/dL, or TC ≥240 mg/dL (20, 21). In addition, given that DM duration data were only available for participants with known DM history, while newly diagnosed patients identified during the survey lacked documented disease duration information, two sensitivity analyses were conducted to evaluate the impact of DM duration on the association between CHG and DR and to demonstrate the robustness of the findings. First, all participants were included, and a DM duration of 0 years was assigned to newly diagnosed patients; DM duration was then incorporated into multivariable logistic regression models. Second, the analysis was restricted to participants with known DM history only, and DM duration was adjusted for. Furthermore, we calculated the E-value to assess the potential impact of unknown confounders on the observed association between CHG and DR (22).

A stratified binary logistic regression model was used to conduct subgroup analyses for multiple categories, including age, sex, WHR, hyperuricemia (HUA), SBP, and DBP. Continuous variables such as age and blood pressure levels were classified based on clinical cut-off points. Specifically, SBP was categorized as <140 mmHg and ≥140mmHg; DBP as <90mmHg and ≥90mmHg (23); Age was divided into four groups: <50 years, 50–60 years, 60–70 years, and ≥70 years. High WHR levels were defined as ≥0.90 for males and ≥0.85 for females (24); HUA was defined as ≥360μmol/L for females and ≥420μmol/L for males (25). The model adjusted for DBP, BMI, age, WHR, sex, HbA1c, SBP, HIP, and TG, but did not include the stratification factors. P-values for multiple subgroup comparisons were adjusted using the Bonferroni correction, with an adjusted significance threshold of α = 0.008 (0.05/6). Potential interactions were evaluated using likelihood ratio tests by comparing models with and without interaction terms.

Finally, receiver operating characteristic (ROC) curves were constructed to analyze the discriminatory ability of CHG for DR and to compare it with other commonly used insulin resistance (IR) indices, including TyG, TyG-BMI, and THR, as well as FPG and HbA1c. The statistical significance of differences between ROC curves was assessed using DeLong’s test. The corresponding area under the curve (AUC), best thresholds, sensitivity, and specificity were also calculated.

The study results were reported in accordance with the STROBE guidelines (26). Data analyses were conducted with R (version 3.4.3) and Empower (version 4.2). For all analyses, a two-sided P-value below 0.05 was regarded as statistically significant.

## Results

### Demographic characteristics of participants

**Figure 2:**
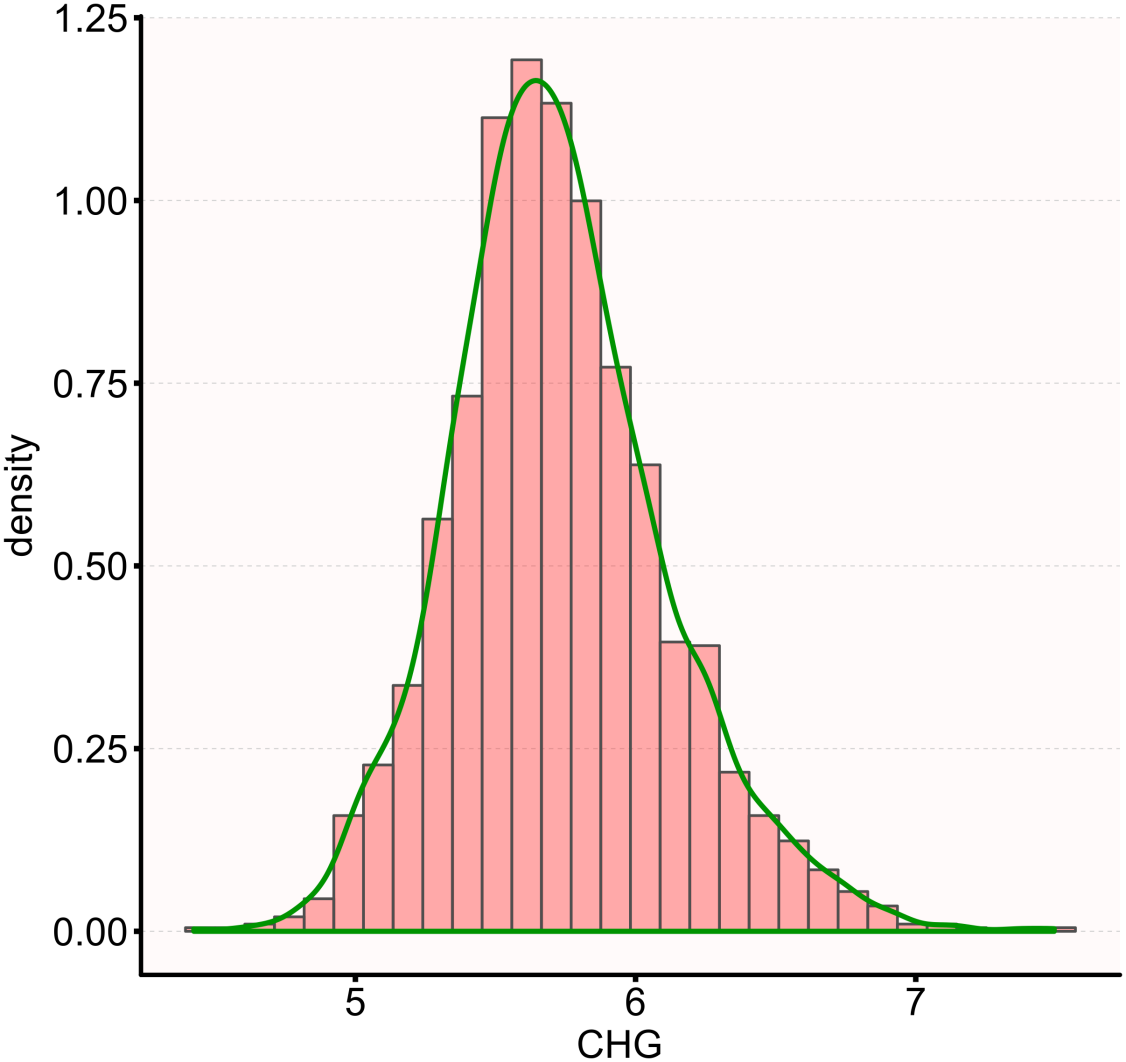
Distribution of CHG. The distribution appeared normal, spanning from 4.42 to 7.49, with a mean ± SD of 5.73 ± 0.38.

**Table 1** lists the demographic and clinical characteristics of 1,909 participants with DM, with males comprising 34.36% of the study population. The CHG values were normally distributed, ranging from 4.42 to 7.49, with a mean (± SD) of 5.73 (± 0.38) (**Figure 2**). Participants were divided into four groups based on the CHG quartiles: Q1 (<5.48), Q2 (5.48-5.69), Q3 (5.69-5.94), and Q4 (≥5.94). Compared to Q1, the higher quartile groups showed higher levels of 2h-PG, FPG, HbA1c, UA, TG, SBP, DBP, weight, BMI, WC, TC, WHR, TyG-BMI, TyG, and LDL-c, while HDL-c levels were relatively lower. Additionally, the proportion of males in the higher quartile groups was greater than that in Q1.

**Table 1:** Baseline characteristics of participants by CHG quartiles.

CHG quartiles	Q1 (<5.48)	Q2 (5.48-5.69)	Q3 (5.69-5.94)	Q4 (≥5.94)	P-value
N	477	477	477	478	
Demographic characteristics					
Age (years)	60.87 ± 8.39	60.42 ± 7.42	60.91 ± 7.84	60.42 ± 8.16	0.654
Sex					<0.001
Male	135 (28.30%)	143 (29.98%)	180 (37.74%)	198 (41.42%)	
Female	342 (71.70%)	334 (70.02%)	297 (62.26%)	280 (58.58%)	
Anthropometric parameters					
Height (m)	1.61 ± 0.08	1.61 ± 0.08	1.62 ± 0.08	1.63 ± 0.09	<0.001
Weight (kg)	66.90 ± 10.65	70.74 ± 11.47	71.54 ± 10.76	71.54 ± 11.39	<0.001
BMI (kg/m²)	25.87 ± 3.58	27.13 ± 3.58	27.06 ± 3.27	26.95 ± 3.40	<0.001
Waist (cm)	91.06 ± 9.83	93.19 ± 9.06	94.08 ± 8.78	94.52 ± 9.04	<0.001
WHR	0.90 ± 0.07	0.91 ± 0.06	0.91 ± 0.06	0.92 ± 0.06	<0.001
HIP (cm)	101.08 ± 7.34	102.47 ± 7.07	103.25 ± 6.68	102.38 ± 6.90	<0.001
Glycemic parameters					
FPG (mg/dL)	114.90 ± 18.32	130.20 ± 21.86	150.29 ± 28.62	208.82 ± 56.67	<0.001
2h PG (mmol/L)	11.81 ± 3.91	12.43 ± 3.72	14.08 ± 4.45	18.79 ± 6.03	<0.001
HbA1c (%)	6.36 ± 0.71	6.63 ± 0.84	7.08 ± 1.14	8.84 ± 3.03	<0.001
Lipid parameters					
TC (mg/dL)	199.47 ± 38.32	212.23 ± 37.32	217.50 ± 38.83	233.41 ± 47.35	<0.001
TG (mg/dL)	113.96 ± 55.77	156.33 ± 78.75	184.43 ± 120.41	228.71 ± 175.16	<0.001
HDL-c (mg/dL)	58.34 ± 13.40	51.33 ± 10.51	48.35 ± 9.63	46.35 ± 9.46	<0.001
LDL-c (mg/dL)	115.72 ± 31.55	129.01 ± 30.89	131.68 ± 32.60	140.03 ± 37.31	<0.001
Blood pressure					
SBP (mmHg)	145.60 ± 21.47	149.63 ± 20.55	151.15 ± 21.74	149.15 ± 22.07	<0.001
DBP (mmHg)	80.00 ± 11.46	82.56 ± 11.80	83.30 ± 12.36	82.30 ± 11.69	<0.001
Other Parameters					
UA (μmol/L)	310.56 ± 74.42	327.70 ± 68.52	326.53 ± 75.22	318.93 ± 88.05	0.002
Metabolic indices					
TyG	8.67 ± 0.45	9.11 ± 0.41	9.37 ± 0.49	9.86 ± 0.59	<0.001
TyG-BMI	224.68 ± 35.42	247.26 ± 35.68	253.62 ± 34.84	265.78 ± 37.48	<0.001
THR	1.80 (1.27-2.59)	2.80 (1.89-4.05)	3.20 (2.26-4.86)	4.06 (2.70-6.10)	<0.001

Values are presented as mean ± standard deviation (SD), median (interquartile range), or number (%).

TG, triglycerides; WHR, waist-to-hip ratio; FPG, fasting plasma glucose; LDL-c, low-density lipoprotein cholesterol; HbA1c, hemoglobin A1c; SBP, systolic blood pressure; TyG, triglyceride-glucose index; UA, uric acid; BMI, body mass index; THR, triglyceride-HDL-cholesterol ratio; TC, total cholesterol; DBP, diastolic blood pressure; HDL-c, high-density lipoprotein cholesterol; 2h PG, 2-hour postprandial glucose; CHG, total cholesterol, high-density lipoprotein, and glucose index; TyG-BMI, triglyceride-glucose-body mass index.

### The prevalence of DR

**Table 2** presents the prevalence of DR among individuals living with DM. The results indicated that 230 participants were diagnosed with DR, resulting in an overall prevalence rate of 12.05%. Specifically, the prevalence rates of DR for the CHG quartiles were as follows: 6.08%(Q1), 7.34% (Q2), 12.37% (Q3), and 22.38% (Q4).

**Table 2:** Prevalence rate of DR (%).

CHG quartiles	Participants(n)	DR events(n)	Prevalence rate (95% CI) (%)
Total	1909	230	12.05(10.59-13.51)
Q1(<5.48)	477	29	6.08(3.93-8.23)
Q2 (5.48-5.69)	477	35	7.34(4.99-9.69)
Q3 (5.69-5.94)	477	59	12.37(9.40-15.33)
Q4 (≥5.94)	478	107	22.38(18.63-26.14)
P for trend			<0.001

DR, diabetic retinopathy; CHG, total cholesterol, high-density lipoprotein, and glucose index; CI, confidence interval; n, number.

**Figure 3:**
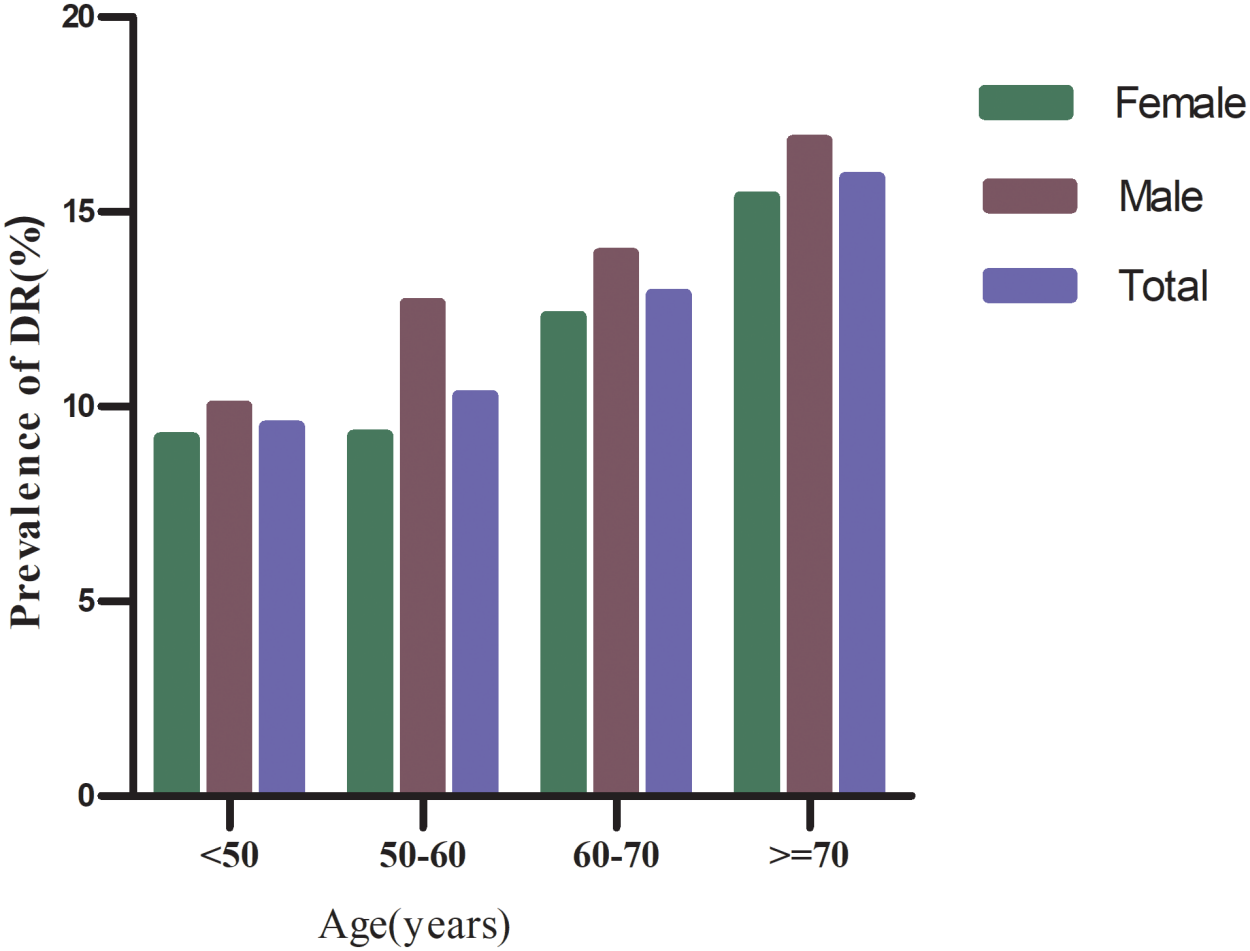
Prevalence of DR (%) stratified by 10-year age groups and sex.

Based on the following age stratification: under 50 years, 50–60 years, 60–70 years, and 70 years and older, the prevalence of DR among males was higher than that of females in all age groups (**Figure 3**). Additionally, the prevalence of DR among both males and females increased with age.

### The relationship between CHG and DR

To explore the association between CHG and DR, two binary logistic regression models were established. Model I (unadjusted) indicated that for every 0.1-unit increase in CHG, there was a significant association with DR (OR = 1.166, 95% CI: 1.126-1.208), corresponding to a 16.6% increase in DR prevalence. In Model II, after considering a comprehensive set of potential confounding factors (age, sex, BMI, WHR, SBP, DBP, HbA1c, HIP, and TG), the association remained significant, with a 0.1 unit increase in CHG associated with a 14.2% increase in DR prevalence (OR = 1.142, 95% CI: 1.081-1.206). Model III was a fully adjusted model using GAM that incorporated continuous covariates (age, BMI, WHR, SBP, DBP, HbA1c, HIP, and TG) as smooth functions, with sex as a categorical variable. The results from Model III were consistent with those of the fully adjusted Model II. Specifically, for every 0.1 unit increase in CHG, the association with DR remained statistically significant (OR = 1.094, 95% CI: 1.033-1.159), representing 9.4% higher odds of DR (**Table 3**).

**Table 3:** Association between CHG and DR in different models.

Exposure	Model I(OR,95%CI) p-value	Model II OR,95%CI) p-value	Model III OR,95%CI) p-value
CHG (per 0.1-unit)	1.166 (1.126, 1.208) <0.001	1.142 (1.081, 1.206) <0.001	1.094 (1.033, 1.159) 0.002
CHG quartiles			
Q1	Ref	Ref	Ref
Q2	1.223 (0.735, 2.036) 0.438	1.235 (0.735, 2.076) 0.425	1.184 (0.695, 2.015) 0.535
Q3	2.180 (1.371, 3.468) <0.001	1.960 (1.205, 3.186) 0.007	1.644 (0.982, 2.753) 0.059
Q4	4.455 (2.890, 6.868) <0.001	2.980 (1.747, 5.083) <0.001	2.140 (1.203, 3.807) 0.009
P for trend	<0.001	<0.001	<0.001

Model I: we did not adjust other covariates.

Model II: we adjust DBP, BMI, age, WHR, sex, HbA1c, SBP, HIP, and TG.

Model III: we adjust DBP (smooth), BMI (smooth), age (smooth), WHR (smooth), sex, HbA1c (smooth), SBP (smooth), HIP (smooth), and TG (smooth).

CHG, total cholesterol, high-density lipoprotein, and glucose index; DR, diabetic retinopathy; OR, odds ratio; CI, confidence interval; Ref, reference; DBP, diastolic blood pressure; BMI, body mass index; WHR, waist-to-hip ratio; HbA1c, hemoglobin A1c; SBP, systolic blood pressure; HIP, hip circumference; TG, triglycerides.

In addition, the categorical variable classified based on the quartiles of CHG was reintroduced into the binary logistic regression model for analysis. Using the lowest quartile (Q1) as the reference group, the multivariable-adjusted ORs for the association between CHG and DR were 1.235 (95% CI: 0.735-2.076) for the Q2 group, 1.960 (95% CI: 1.205-3.186) for the Q3 group, and 2.980 (95% CI: 1.747-5.083) for the Q4 group. These results indicate that compared to Q1, Q2 showed a 24% increase in DR prevalence with no statistical significance, while Q3 and Q4 showed significantly higher DR prevalence, with increases of 96% and 198%, respectively (**Table 3**, Model II).

### Sensitivity analysis

To assess the stability of our study results, various sensitivity analyses were conducted. Initially, the sample was limited to participants with TG levels below 150.45 mg/dL (Model I). After adjusting for confounding factors (DBP, BMI, age, WHR, sex, HbA1c, SBP, and HIP), it was found that for every 0.1-unit increase in CHG, there were 11.4% higher odds of DR (OR = 1.114, 95% CI: 1.033-1.202). Among individuals with a BMI < 28 kg/m²(Model II), after adjusting for covariates including DBP, TG, age, WHR, sex, HbA1c, SBP, and HIP, the positive association between CHG (per 0.1-unit increase) and DR remained statistically significant (OR = 1.156, 95% CI: 1.087-1.230). Finally, when the sample was restricted to participants with TC < 240 mg/dL (Model III), after adjusting for covariates including DBP, TG, age, WHR, sex, HbA1c, SBP, BMI, and HIP, similar results were observed, with an OR of 1.152 (95% CI: 1.088-1.220) for each 0.1-unit increase in CHG (**Table 4**).

**Table 4:** Association between CHG and DR evaluated across multiple sensitivity analyses.

Exposure	Model I(OR,95%CI) p-value	Model II(OR,95%CI) p-value	Model III(OR,95%CI) p-value
CHG (per 0.1-unit)	1.114 (1.033, 1.202) 0.005	1.156 (1.087, 1.230) <0.001	1.152 (1.088, 1.220) <0.001
CHG quartiles			
Q1	Ref	Ref	Ref
Q2	1.219 (0.673, 2.209) 0.514	1.331 (0.730, 2.428) 0.350	1.553 (0.890, 2.710) 0.121
Q3	2.187 (1.238, 3.863) 0.007	2.273 (1.303, 3.966) 0.004	2.574 (1.513, 4.379) <0.001
Q4	2.297 (1.104, 4.779) 0.026	2.885 (1.535, 5.421) 0.001	4.170 (2.290, 7.594) <0.001

Model I was a sensitivity analysis in participants with TG<150 mg/dL. Adjusted DBP, BMI, age, WHR, sex, HbA1c, SBP, HIP, and TG.

Model II was a sensitivity analysis in participants with BMI<28 kg/m². Adjusted DBP, BMI, age, WHR, sex, HbA1c, SBP, HIP, and TG.

Model III was a sensitivity analysis in participants with TC<240 mg/dL. Adjusted DBP, BMI, age, WHR, sex, HbA1c, SBP, HIP, and TG.

CHG, total cholesterol, high-density lipoprotein, and glucose index; DR, diabetic retinopathy; OR, odds ratio; CI, confidence interval; Ref, reference; DBP, diastolic blood pressure; BMI, body mass index; WHR, waist-to-hip ratio; HbA1c, hemoglobin A1c; SBP, systolic blood pressure; HIP, hip circumference; TG, triglycerides.

In addition, to address potential confounding by DM duration, two sensitivity analyses were performed (**Supplementary Table S2**). In Model I, all participants (n = 1,909) were included, with newly diagnosed patients assigned a DM duration of 0 years. After adjusting for other covariates plus DM duration, each 0.1-unit increase in CHG was associated with 10.8% higher odds of DR (OR = 1.108, 95% CI: 1.051–1.167, P < 0.001). In Model II, the analysis was restricted to participants with known DM duration only (n = 790), with actual DM duration and other covariates adjusted for. The association remained significant, with each 0.1-unit increase in CHG associated with 7.2% higher odds of DR (OR = 1.072, 95% CI: 1.018–1.129, P = 0.009). These consistent findings across both analytical approaches demonstrated that the association between CHG and DR was robust and independent of DM duration.

Moreover, an E-value of 1.54 was calculated, which was greater than the relative risk (1.37) of the association between CHG and potential unmeasured confounding factors but less than the relative risk (1.85) of the association between unmeasured confounding factors and DR. This indicated that unknown or unmeasured confounding factors were unlikely to have a significant impact on the relationship between CHG and DR. These sensitivity analyses enhanced the reliability and stability of our results.

### Subgroup analysis

In the pre-defined and exploratory subgroup analyses (**Table 5**), the associations between CHG and DR were evaluated across various subgroups stratified by age, sex, WHR, HUA, SBP, and DBP. After Bonferroni correction for 6 multiple comparisons (adjusted α=0.008), the positive associations between CHG and DR remained statistically significant across nearly all subgroups (all adjusted P < 0.05), with the exception of participants aged <50 years (OR = 1.108, 95% CI 0.960-1.278, p=0.1609), likely due to the limited sample size (n=137) and lower event rate in this age group. No significant interactions were detected between CHG and the stratification variables (all P for interaction ≥ 0.05), indicating that these factors did not significantly modify the association between CHG and DR.

**Table 5:** Stratified relationship between CHG and DR by age, WHR, HUA, SBP, sex, and DBP.

Characteristics	Participants	OR (95% CI)	P value	P for interaction
Age (years)				0.531
<50	137	1.108 (0.960, 1.278)	0.161	
50-60	769	1.180 (1.095, 1.272)	<0.001	
60-70	707	1.110 (1.032, 1.194)	0.005	
≥70	296	1.138 (1.043, 1.242)	0.004	
Sex				0.415
Male	656	1.163 (1.082, 1.250)	<0.001	
Female	1253	1.128 (1.060, 1.199)	<0.001	
WHR				0.980
Low	797	1.149 (1.069, 1.235)	<0.001	
High	1112	1.148 (1.081, 1.220)	<0.001	
HUA				0.193
No	1799	1.141 (1.080, 1.205)	<0.001	
Yes	110	1.265 (1.079, 1.485)	0.004	
SBP (mmHg)				0.917
<140	695	1.141 (1.058, 1.230)	0.001	
≥140	1241	1.145 (1.079, 1.215)	<0.001	
DBP (mmHg)				0.557
<90	1442	1.135 (1.072, 1.202)	<0.001	
≥90	467	1.165 (1.066, 1.274)	0.001	

Note 1: Above model adjusted for DBP, BMI, age, WHR, sex, HbA1c, SBP, HIP, and TG.

Note 2: In each case, the above model is not adjusted for the stratification variable.

Note 3: P-values were adjusted using Bonferroni correction for 6 multiple comparisons (adjusted α = 0.008).

CHG, total cholesterol, high-density lipoprotein, and glucose index; DR, diabetic retinopathy; OR, odds ratio; CI, confidence interval; Ref, reference; DBP, diastolic blood pressure; BMI, body mass index; HbA1c, hemoglobin A1c; SBP, systolic blood pressure; HIP, hip circumference; HUA, hyperuricemia; TG, triglycerides.

### ROC analysis of the discriminative ability of CHG, TyG-BMI, TyG, THR, FPG, and HbA1c for identifying DR

**Figure 4:**
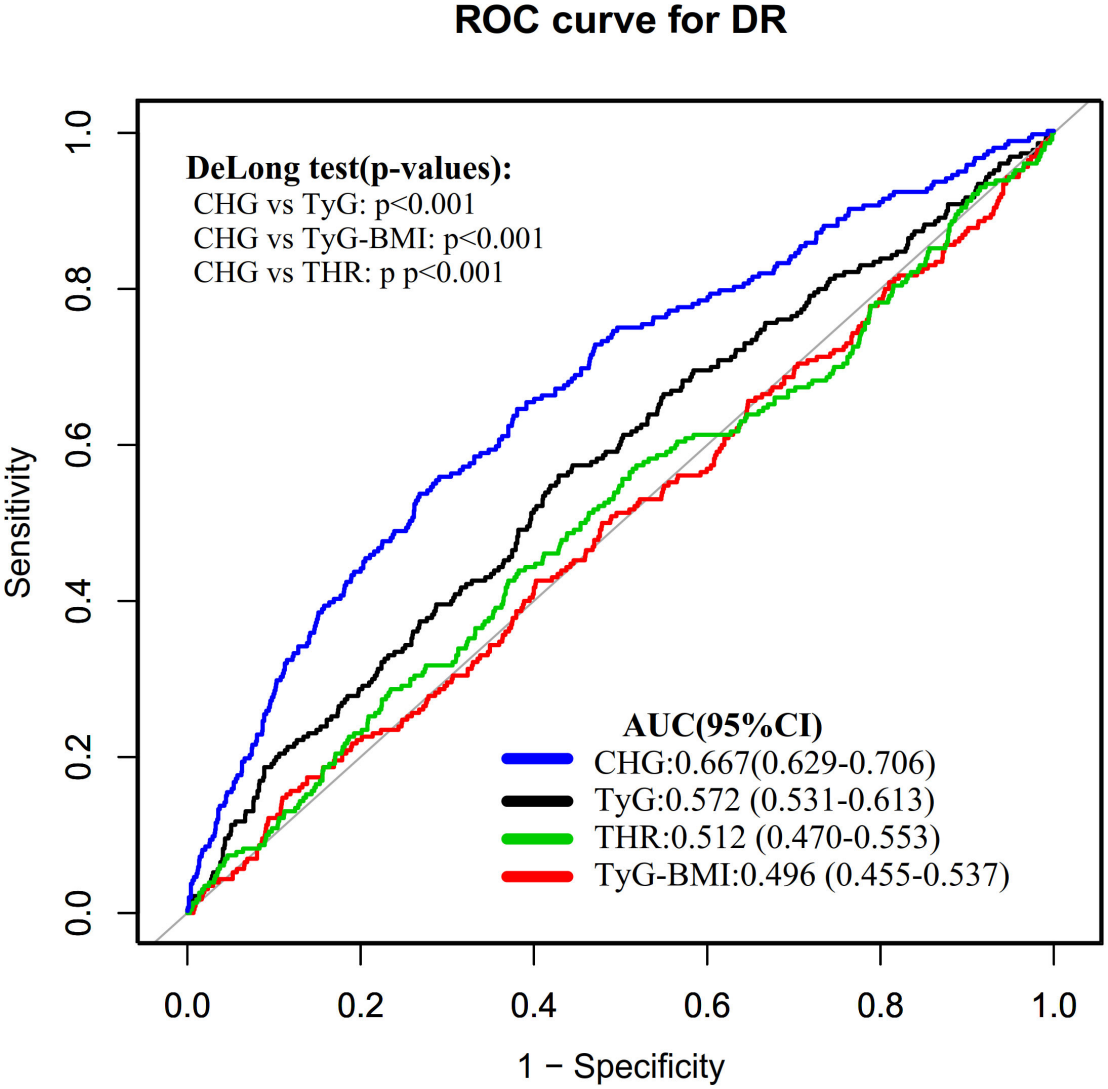
ROC curves of CHG, TyG-BMI, TyG, and THR for the discrimination of DR.

The ROC curve was plotted to evaluate the discriminative ability of CHG, TyG-BMI, TyG, and THR for identifying DR (**Figure 4**). The AUC values for each variable were as follows: 0.4958 (0.4548–0.5368) < THR: 0.5118 (0.4704–0.5532) < TyG: 0.5720 (0.5314–0.6125) < CHG: 0.6673 (0.6287–0.7059). The Youden index values for TyG, TyG-BMI, THR, and CHG were 0.1324, 0.0381, 0.0567, and 0.2668, respectively. This indicates that CHG has the highest Youden index and AUC compared to TyG-BMI, TyG, and THR, demonstrating the strongest discriminative ability for identifying DR (**Table 6**).

**Table 6:** The discriminative ability of CHG, TyG-BMI, TyG, and THR for DR.

Test	AUC (95% CI)	Best threshold	Specificity	Sensitivity	Youden index
TyG	0.572 (0.531-0.613)	9.300	0.572	0.561	0.132
TyG-BMI	0.496 (0.455-0.537)	202.615	0.890	0.148	0.038
THR	0.512 (0.470-0.553)	2.379	0.618	0.439	0.057
CHG	0.667 (0.629-0.706)	5.881	0.732	0.535	0.267

CHG, cholesterol, high-density lipoprotein cholesterol, glucose index; TyG-BMI, triglyceride-glucose-body mass index; TyG, triglyceride-glucose index; THR, triglyceride-HDL-cholesterol ratio; DR, diabetic retinopathy; AUC, area under the curve; CI, confidence interval.

Additionally, ROC curves were plotted to evaluate and compare the discriminatory ability of FPG, HbA1c, and CHG in identifying DR (**Supplementary Figure S1**). The AUC (95% CI) values for each variable were as follows: FPG: 0.631 (0.596–0.657), CHG: 0.667 (0.629–0.706), and HbA1c: 0.679 (0.623–0.713). Comparison of ROC curves using DeLong’s test revealed no significant difference between CHG and HbA1c in their ability to discriminate DR (**Supplementary Table S2**, **Supplementary Figure S1**).

## Discussion

This study indicated that there was an independent and positive association between CHG and DR. Furthermore, the ROC curve analysis showed that CHG was superior to traditional IR indicators in discriminating DR.

IR is characterized by a reduction in the biological efficacy of insulin and is a key pathological factor in the development of various metabolic diseases such as obesity, metabolic syndrome, DM, and non-alcoholic fatty liver disease (27–30). It is considered the primary pathophysiological basis for DM and its complications (31–33). Currently, alternative markers used to assess IR include TyG-BMI, TyG, and THR, which have garnered attention due to their correlation with metabolic diseases such as DM, cardiovascular disease, stroke, and hypertension (15, 34–38). In recent years, a novel metabolic disorder marker that integrates glucose and lipid levels—the CHG index—has been shown to significantly outperform traditional IR indicators in discriminating DM (10). Specifically, compared to TyG-BMI (AUC: 0.698) and TyG (AUC: 0.825), CHG (AUC: 0.864) has the highest AUC in predicting DM (10). Additionally, CHG also demonstrates significant value in the assessment of cardiovascular disease (11). However, current literature on the relationship between CHG and DR remains quite limited. There is only one cross-sectional study involving 175 individuals with DM that found a significantly higher CHG level among patients with DR (12). The CHG level in the DR group was 2.57 ± 0.24, compared to 2.47 ± 0.20 in the non-DR group (P = 0.008). However, the multivariable logistic regression analysis did not reveal a significant association between CHG and DR (12). Our study showed that elevated CHG levels were significantly and positively associated with DR. This finding differs from the previous study for several reasons. First, there is a difference in sample size; compared to the earlier small sample study, our research has a larger sample size, providing greater statistical power. Secondly, the prior study predominantly focused on individuals with DM who had a disease duration of more than 10 years, whereas many individuals may already exhibit pathological changes of DR at the time of DM diagnosis (39). Therefore, our study population is not restricted by the duration of DM, including all patients with DM. In addition, differences in sex distribution, study time periods, and adjustment factors may also contribute to these discrepancies. Furthermore, we analyzed CHG as both a categorical and continuous variable to minimize information loss and more accurately quantify its association with outcome variables. To further ensure the reliability of the study results, we conducted a sensitivity analysis on participants with a BMI <28 kg/m², TG < 150.45 mg/dL, and TC < 240 mg/dL. The results confirmed the consistency of these findings within these specific subgroups. In summary, identifying CHG as being associated with DR and clarifying this association is of significant clinical importance. Incorporating CHG into routine clinical assessments could assist healthcare providers in DR identification and patient assessment. As a simple, cost-effective biomarker derived from routine laboratory tests (TC, HDL-c, and FPG), CHG may serve as a valuable screening tool for DR, particularly in resource-limited settings where access to specialized ophthalmologic services is constrained. This index could facilitate screening strategies, enabling clinicians to prioritize patients who require more intensive monitoring or earlier ophthalmologic referrals.

Additionally, ROC analysis was performed, which revealed that the discriminative ability of CHG for identifying DR was higher compared to other commonly used IR indicators (TyG-BMI, TyG, and THR), with an AUC value of 0.667. This finding suggests that CHG, as a novel, clinically accessible, and reproducible indicator, holds some discriminative ability in assessing DR. Future multi-center, large-sample cohort studies should compare CHG with established insulin resistance indices (e.g., Homeostatic Model Assessment for Insulin Resistance, HOMA-IR) and metabolic syndrome diagnostic criteria to validate its generalizability and clinical reliability, thereby providing an important reference for DR assessment. ROC analysis also demonstrated that CHG exhibited comparable discriminative ability for DR to HbA1c, while outperforming FPG alone. Although HbA1c is widely used as a common indicator for assessing long-term glycemic control and identifying diabetic complications, it requires specialized laboratory testing, which is difficult to implement in resource-limited settings. In contrast, CHG can be calculated directly from routine fasting lipid and glucose test data, offering greater accessibility and cost-effectiveness. Moreover, HbA1c reflects only glycemic status over the preceding 2–3 months and cannot capture lipid metabolism abnormalities, whereas CHG integrates glucose and lipid parameters into a composite indicator that reflects the multifactorial metabolic disturbances underlying DR pathogenesis. Therefore, CHG may serve as a complement to HbA1c in clinical practice for DR discrimination, particularly in primary care settings or populations where standard HbA1c testing is unavailable.

The observed association between CHG and DR may be explained by several interconnected pathophysiological mechanisms. First, the combined effects of hyperglycemia and hypertriglyceridemia synergistically impair retinal vascular endothelial function through activation of damaging pathways, including advanced glycation end-product (AGE) formation, protein kinase C (PKC) activation, and inflammatory responses, ultimately compromising blood-retinal barrier integrity (40–42). Second, both elevated glucose and triglycerides independently promote excessive reactive oxygen species (ROS) production in retinal tissue—hyperglycemia via mitochondrial superoxide generation and hypertriglyceridemia through increased fatty acid β-oxidation—triggering retinal capillary cell apoptosis, pericyte loss, and pathological neovascularization (43, 44). Third, low HDL-cholesterol levels compromise the protective effects of HDL on retinal microvasculature, including its anti-inflammatory, antioxidant, and cholesterol efflux functions, thereby increasing vulnerability to metabolic injury (45, 46). Fourth, the glucose-lipid metabolic crosstalk creates a vicious cycle wherein hyperglycemia promotes lipid accumulation (lipotoxicity) in retinal cells while dyslipidemia exacerbates insulin resistance, amplifying retinal microvascular damage (47, 48). By simultaneously integrating glucose, TC, and HDL-c, the CHG index captures this multifactorial metabolic stress, providing biological plausibility for its superior discriminative ability in identifying DR.

It is important to acknowledge that our study explored the association between CHG and clinically detectable DR. However, the paradigm of DR management is evolving toward earlier identification and intervention, targeting stages even before clinical manifestations become apparent. The emerging concept of “functional DR” recognizes retinal dysfunction that precedes fundus-visible microvascular changes, characterized by early neurovascular and metabolic disturbances (49). Given that metabolic stress, reflected by indicators such as CHG, precedes visible microvascular lesions, the CHG index may demonstrate discriminative ability for functional or subclinical DR. Unfortunately, the current analysis cannot directly reveal the association between CHG and different stages of DR progression, from early functional changes to advanced clinically detectable stages. Future prospective studies utilizing advanced diagnostic modalities—such as electroretinography, retinal function imaging, or high-resolution optical coherence tomography—are warranted to verify whether CHG is associated with the onset or progression of functional DR as well as its relationship with later clinical stages (6, 50, 51). Such investigations would provide important evidence for early screening strategies and individualized intervention in the pre-clinical phase of DR.

This study has several important advantages: (i) To our knowledge, this is the first large-sample study to explore the association between CHG and DR. CHG was analyzed both as a continuous variable and by dividing it into quartiles, which minimizes information loss and enables us to assess its association with DR more comprehensively and accurately. (ii) ROC curve analysis showed CHG demonstrated better discriminative ability for DR than other IR indicators, suggesting potential utility as a screening tool. (iii) Multiple imputation methods were employed to handle missing data, thereby enhancing statistical power and reducing bias caused by the absence of information on covariates. (iv) A series of sensitivity analyses were conducted to validate the findings. These included converting CHG into a categorical variable, incorporating continuous covariates as curves in a GAM, calculating E-values to estimate the potential impact of unmeasured confounders, and re-evaluating the association between CHG and DR in populations with BMI below 28kg/m², TG < 150.45 mg/dL, and TC < 240 mg/dL.

However, this study has certain limitations that need to be acknowledged. First, the study subjects were limited to the Chinese population. This limitation restricts the generalizability of the results to different ethnic groups or geographical regions; therefore, further validation in more heterogeneous populations is needed. Second, this was a cross-sectional study that observed an independent association between CHG and DR. Future research should further validate the relationship between them through multicenter, large-sample cohort studies. In addition, since this study was a secondary analysis, some important potential confounders were not included in the multivariable regression models, including renal function parameters, anemia indices, and lifestyle factors. Although we calculated an E-value, which suggested that these unknown or unmeasured confounding factors were unlikely to have a significant impact on the association between CHG and DR, this sensitivity analysis still cannot completely replace the necessity of directly incorporating these specific clinically important confounders. In future research, whether conducted independently or in collaboration with other research institutions, more relevant variables should be included, such as renal function indicators, anemia indices, and detailed lifestyle information (smoking, drinking, diet, and physical activity), to achieve a more comprehensive analysis of the association between CHG and DR and to further validate the findings of this study. Fourth, our study only classified DR as a binary outcome (presence vs. absence) based on fundus photography, without detailed severity grading according to standardized scales such as the International Clinical Diabetic Retinopathy Severity Scale. This precluded examining whether the CHG-DR association varies across different stages of retinopathy progression. Future studies with standardized DR grading are needed to validate these findings. Finally, as an observational study, while an independent association between CHG and DR was observed, causality cannot be established.

## Conclusion

This study found a significant independent positive association between CHG and DR. Furthermore, compared to traditional indicators of IR, including TyG-BMI, TyG, and THR, CHG demonstrates stronger discriminative ability for identifying DR. This provides a new perspective for DR assessment in clinical practice. Future multi-center, prospective cohort studies are needed to further elucidate the temporal relationship between CHG and DR, evaluate its performance against established IR indices like HOMA-IR and metabolic syndrome indices, and confirm generalizability across diverse populations.

## Data Availability

The datasets presented in this study can be found in online repositories. The names of the repository/repositories and accession number(s) can be found below: https://journals.plos.org/plosone/article?id=10.1371/journal.pone.0166597.
