# Supplementary figures and images for: Total cholesterol, high-density lipoprotein, and glucose (CHG) index and diabetic retinopathy in middle-aged and elderly Chinese adults with diabetes: a cross-sectional study
Source: Front Endocrinol (Lausanne). 2026 Jan 22;16:1682279. doi: 10.3389/fendo.2025.1682279 (PMC12872500; doi:10.3389/fendo.2025.1682279)

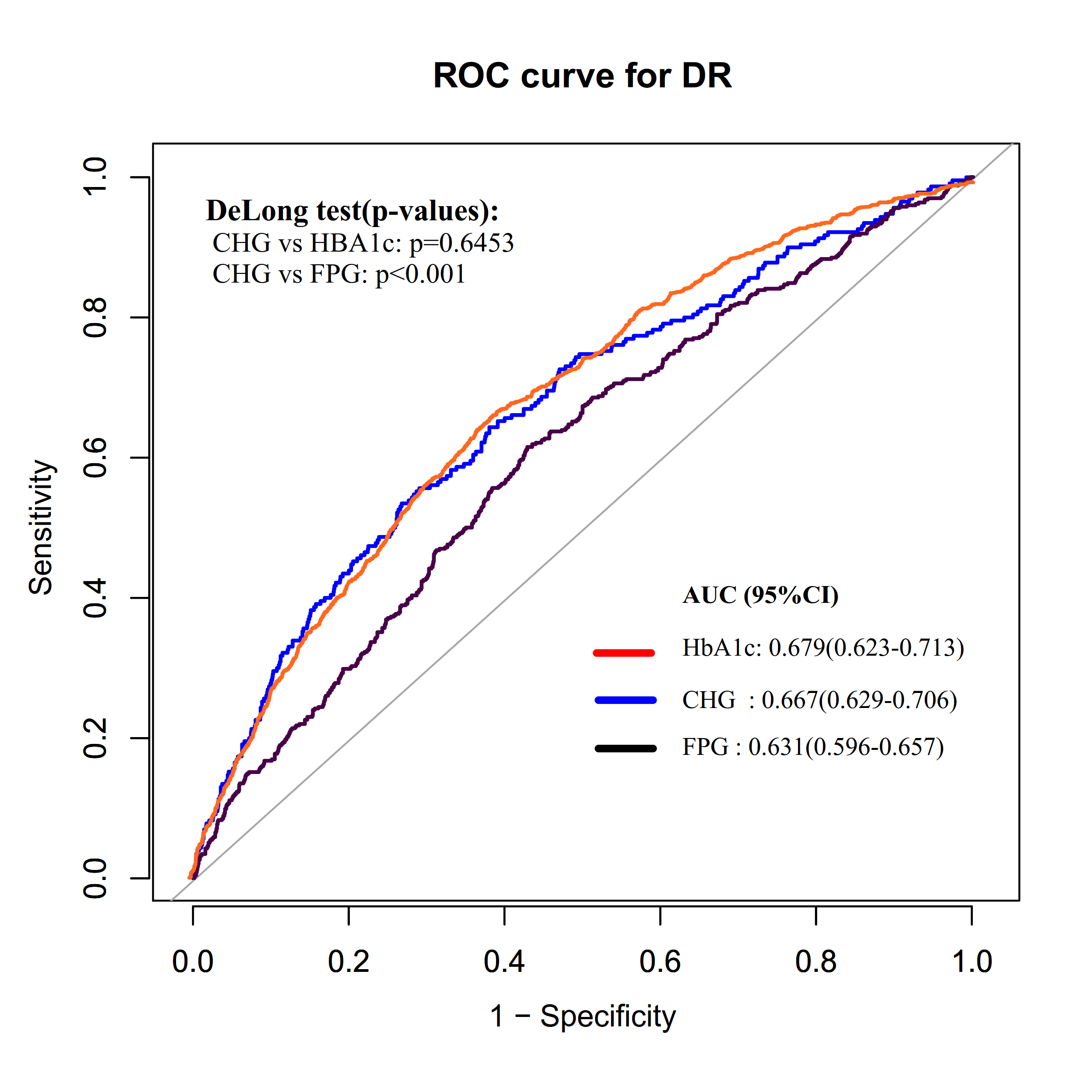

Supplement: Supplementary file 1 [file Image1.tif]
