# Supplementary material for: Total cholesterol, high-density lipoprotein, and glucose (CHG) index and diabetic retinopathy in middle-aged and elderly Chinese adults with diabetes: a cross-sectional study
Source: Front Endocrinol (Lausanne). 2026 Jan 22;16:1682279. doi: 10.3389/fendo.2025.1682279 (PMC12872500; doi:10.3389/fendo.2025.1682279)
Supplement: Supplementary file 2 [file Table1.docx]

**Total Cholesterol, High-Density Lipoprotein, and Glucose (CHG) Index and Diabetic Retinopathy in Middle-Aged and Elderly Chinese Adults with Diabetes: A Cross-Sectional Study**

**Running title: CHG and** **Diabetic Retinopathy**

**Yingpin Cao^1,2#^, Yuqin He^3^, Jiaqian Zhu^4*^, Yong Han^5*^**

^1^Hunan Provincial University Key Laboratory of the Fundamental and Clinical Research on Neurodegenerative Diseases, Changsha Medical University, Changsha, 410219, Hunan, China

^2^Department of Ophthalmology, Shenzhen People’s Hospital (The First Affiliated Hospital, Southern University of Science and Technology; The Second Clinical Medical College, Jinan University) Shenzhen, 518020, Guangdong Province, China.

^3^Hunan Provincial University Key Laboratory of the Fundamental and Clinical Research on Neurodegenerative Diseases, School of Medical Imaging, Changsha Medical University, Changsha, 410219, Hunan Province, China.

^4^Department of Neurology, The First Affiliated Hospital of Shenzhen University, Shenzhen Second People's Hospital, Shenzhen University, Shenzhen 518035, Guangdong Province, China.

^5^Department of Emergency, Shenzhen Second People's Hospital, The First Affiliated Hospital of Shenzhen University, Shenzhen 518035, Guangdong Province, China.

***Corresponding author**

Jiaqian Zhu

Department of Neurology, Shenzhen Second People's Hospital

No.3002 Sungang West Road, Futian District,

Shenzhen 518035,

Guangdong Province,

China.

Email: zhujiaqian1122@163.com

***Corresponding author**

Yong Han

Department of Emergency, Shenzhen Second People's Hospital

No.3002 Sungang West Road, Futian District,

Shenzhen 518035,

Guangdong Province,

China.

Email: Hanyong511023@163.com

**Table S1 Collinearity screening**

|  | Step 1 | Step 2 | Step 3 | Step 4 |
| --- | --- | --- | --- | --- |
| CHR | 2.9 | 2.9 | 2.6 | 2.6 |
| Age (years) | 1.3 | 1.3 | 1.3 | 1.3 |
| SEX | 2.1 | 2.1 | 2.1 | 2.1 |
| WHR | 3.1 | 3.1 | 3.1 | 1.2 |
| HBA1c (%) | 1.6 | 1.6 | 1.6 | 1.6 |
| Height (m) | 63.4 | 2.5 | 2.5 | 2 |
| Weight (Kg) | 149.7 | NA | NA | NA |
| BMI(m^2^/kg) | 100.9 | 3.5 | 3.5 | 1.2 |
| Waist(cm) | 6.8 | 6.8 | 6.8 | NA |
| HDL-c (mg/dL) | 2.5 | 2.5 | 1.7 | 1.7 |
| LDL-c (mg/dL) | 6.2 | 6.2 | 1.5 | 1.5 |
| TG(mg/dL) | 2.6 | 2.6 | 1.5 | 1.5 |
| UA(μmol/L) | 1.3 | 1.3 | 1.3 | 1.3 |
| SBP(mmHg) | 1.7 | 1.7 | 1.7 | 1.7 |
| DBP(mmHg) | 1.6 | 1.6 | 1.6 | 1.6 |

Variables excluded by collinearity screening: Waist

Note: VIF, variance inflation factor. If the VIF value was greater than 10, indicating multicollinearity with other variables, the variable was excluded

**Table S2 Association Between CHG and DR after adjusting for DM duration.**

| Exposure | Model I(OR,95%CI) p-value | Model II(OR,95%CI) p-value |
| --- | --- | --- |
| CHG (per 0.1-unit) | 1.108 (1.051, 1.167) 0.000 | 1.072 (1.018, 1.129) 0.009 |
| CHG quartiles |  |  |
| Q1 | Ref | Ref |
| Q2 | 1.273 (0.741, 2.186) 0.383 | 1.325 (0.714, 2.458) 0.372 |
| Q3 | 1.814 (1.096, 3.001) 0.020 | 1.621 (0.923, 2.845) 0.092 |
| Q4 | 2.400 (1.382, 4.169) 0.002 | 1.841 (1.034, 3.276) 0.038 |

Model I: All participants (n =1909); newly diagnosed DM were assigned a duration of 0 years. Adjusted for. Adjusted DBP, BMI, age, WHR, sex, HbA1c, SBP, HIP, TG and DM duration.

Model II: Participants with known DM history only (n =790). Adjusted for DBP, BMI, age, WHR, sex, HbA1c, SBP, HIP, TG and DM duration.

**Table S2. The discriminative ability of CHG, FPG, and HbA1c for DR**

| Test | AUC (95%CI) | Best threshold | Specificity | Sensitivity | Youden Index |
| --- | --- | --- | --- | --- | --- |
| CHG | 0.667(0.629-0.706) | 5.881 | 0.732 | 0.535 | 0.267 |
| FPG (mmol/L) | 0.631(0.596-0.657) | 8.105 | 0.635 | 0.575 | 0.210 |
| HbA1c (%) | 0.679(0.623-0.713) | 7.350 | 0.708 | 0.583 | 0.291 |

Abbreviations: CHG, cholesterol, high-density lipoprotein cholesterol, glucose index; DR, diabetic retinopathy; HbA1c, hemoglobin A1c; FPG, fasting plasma glucose; AUC, area under the curve; CI, confidence interval,

**Figure S1. ROC curves of CHG, FPG, and HbA1c for the discrimination of DR.**
